# Nucleolin Overexpression Predicts Patient Prognosis While Providing a Framework for Targeted Therapeutic Intervention in Lung Cancer

**DOI:** 10.3390/cancers14092217

**Published:** 2022-04-29

**Authors:** Ângela Valério-Fernandes, Nuno A. Fonseca, Nélio Gonçalves, Ana F. Cruz, Marta I. Pereira, Ana C. Gregório, Vera Moura, Ana F. Ladeirinha, Ana Alarcão, Joana Gonçalves, Antero Abrunhosa, Joana B. Melo, Lina Carvalho, Sérgio Simões, João N. Moreira

**Affiliations:** 1 CNC—Center for Neurosciences and Cell Biology, Center for Innovative Biomedicine and Biotechnology (CIBB), Faculty of Medicine (Polo I), University of Coimbra, Rua Larga, 3004-504 Coimbra, Portugal; angel.filipa.fernandes@gmail.com (Â.V.-F.); nuno.a.c.fonseca@gmail.com (N.A.F.); neliomotag@gmail.com (N.G.); ana.filipa.cruz@cnc.uc.pt (A.F.C.); marta.dot.isabel@gmail.com (M.I.P.); anaclgregorio@gmail.com (A.C.G.); vdantasmoura@gmail.com (V.M.); ssimoes@ci.uc.pt (S.S.); 2 IIIUC, Institute for Interdisciplinary Research, University of Coimbra, 3030-789 Coimbra, Portugal; 3 TREAT U, SA-Parque Industrial de Taveiro, Lote 44, 3045-508 Coimbra, Portugal; 4 Univ Coimbra-University of Coimbra, CIBB, Faculty of Pharmacy, Pólo das Ciências da Saúde, Azinhaga de Santa Comba, 3000-548 Coimbra, Portugal; 5 CHUC—Coimbra Hospital and University Center, Praceta Prof. Mota Pinto, 3000-075 Coimbra, Portugal; 6 IAP-PM, Institute of Anatomical and Molecular Pathology, Faculty of Medicine, University of Coimbra, 3004-504 Coimbra, Portugal; analadeirinha@gmail.com (A.F.L.); amalarcao@fmed.uc.pt (A.A.); lcarvalho@chuc.min-saude.pt (L.C.); 7 CIBIT/ICNAS, Institute for Nuclear Sciences Applied to Health, University of Coimbra, 3000-548 Coimbra, Portugal; jgoncalves@fmed.uc.pt (J.G.); antero@pet.uc.pt (A.A.); 8 CACC—Clinical Academic Center of Coimbra, Faculty of Medicine, Pólo das Ciências da Saúde, Azinhaga de Santa Comba, University of Coimbra, 3000-548 Coimbra, Portugal; mmelo@fmed.uc.pt; 9 iCBR—Coimbra Institute for Clinical and Biomedical Research, CIBB, Center of Investigation on Environment Genetics and Oncobiology (CIMAGO), Pólo das Ciências da Saúde, Azinhaga de Santa Comba, 3000-548 Coimbra, Portugal

**Keywords:** lung cancer, nucleolin expression, tumor microenvironment, targeted intracellular drug delivery

## Abstract

**Simple Summary:**

Despite the clinical benefit of new anticancer therapies, such as immune checkpoint inhibitors, lung cancer remains the most frequent cause of cancer-related death worldwide, thus supporting the need to develop novel anticancer treatments. Endothelial cells of the tumor-associated vasculature are easily accessible to drugs administered intravenously, besides having greater genetic stability than neoplastic cells and thus lowering the risk of developing drug resistance. In this respect, the identification of alternative targets, and therapeutic strategies, within the tumor vasculature is of high relevance. Accordingly, this work aimed at characterizing nucleolin expression in patient-derived pulmonary carcinomas and further validating nucleolin as a novel target to mediate successful therapeutic interventions against human lung cancers. The highlighted prognostic value of nucleolin points towards the applicability of nucleolin-based targeting strategies against nucleolin^high^ pulmonary carcinomas, present in every disease stage, in a clinical trial setting.

**Abstract:**

Notwithstanding the advances in the treatment of lung cancer with immune checkpoint inhibitors, the high percentage of non-responders supports the development of novel anticancer treatments. Herein, the expression of the onco-target nucleolin in patient-derived pulmonary carcinomas was characterized, along with the assessment of its potential as a therapeutic target. The clinical prognostic value of nucleolin for human pulmonary carcinomas was evaluated through data mining from the Cancer Genome Atlas project and immunohistochemical detection in human samples. Cell surface expression of nucleolin was evaluated by flow cytometry and subcellular fraction Western blotting in lung cancer cell lines. Nucleolin mRNA overexpression correlated with poor overall survival of lung adenocarcinoma cancer patients and further predicted the disease progression of both lung adenocarcinoma and squamous carcinoma. Furthermore, a third of the cases presented extra-nuclear expression, contrasting with the nucleolar pattern in non-malignant tissues. A two- to twelve-fold improvement in cytotoxicity, subsequent to internalization into the lung cancer cell lines of doxorubicin-loaded liposomes functionalized by the nucleolin-binding F3 peptide, was correlated with the nucleolin cell surface levels and the corresponding extent of cell binding. Overall, the results suggested nucleolin overexpression as a poor prognosis predictor and thus a target for therapeutic intervention in lung cancer.

## 1. Introduction

Lung cancer remains the most frequent cause of cancer-related death worldwide for both men and women [1]. Chemotherapeutic approaches rapidly showed limited efficacy due to systemic toxicity and limited therapeutic efficacy [2], prompting the need for developing targeted therapies. The emergence of the tumoral molecular profiling enabled the identification of driver oncogenic mutations, such as in the EGFR, ALK, ROS1, and BRAF genes [3], which, combined with the immunohistochemical screening of programmed death-ligand 1 (PD-L1) levels, established the current era of personalized therapies revolutionizing the care of lung cancer [4]. Yet, clinical trials with anti-PD-1 therapy (KEYNOTE-006) evidenced that, despite the clinical benefit from the responding subgroup (45%), there are still 55 to 67% non-responders [5], thus supporting both heterogeneity and the need to develop novel anticancer treatments. It has also been observed that a substantial number of cancer patients are intrinsically resistant or become refractory under the therapeutic regimens combining standard chemotherapy and targeted agents, especially those driving antiangiogenic effects [6], most likely due to host factors and stromal components [7]. Despite such therapeutic obstacles, tumor endothelial cells remain relevant for targeting strategies as they present higher genetic stability than neoplastic cells, with a lower risk of developing drug resistance [8] and higher accessibility for intravenously administered drugs. Therefore, identifying other targets within the tumor vasculature and developing matching therapeutic strategies may be the next step to impair lung cancer progression.

Nucleolin is a nucleolar protein involved in several biological functions, including chromatin structure, ribosome assembly, nucleus–cytoplasm transport, and cell cycle progression, as well as self-renewal maintenance of embryonic stem cells [9,10]. Cell surface nucleolin has been reported as a target for anticancer therapies in tumors other than lung cancer [11,12,13] as it is overexpressed in cancer cells and endothelial cells from tumor blood vessels [14]. We have previously demonstrated a nucleolin enrichment in breast cancer cells as nucleolin-binding F3-peptide-targeted pH-sensitive pegylated liposomes were significantly internalized by breast cancer cells [15], particularly by cancer stem cells subpopulations [16], in a ligand-dependent manner, in contrast with the absence of internalization by human non-cancer fibroblasts [17]. Additionally, the aforementioned F3-peptide-targeted pH-sensitive liposomes containing doxorubicin (Dox; used as a model drug) efficiently decreased both the tumor viable rim area and microvascular density, limiting tumor invasion in nucleolin-overexpressing MDA-MB-435S-derived tumors implanted in the mammary fat pad of female Balb/c nude mice [18]. Furthermore, using a GMP-grade version of those F3-peptide-targeted liposomes (named PEGASEMP), it was demonstrated that shifting from the cancer-cell-targeting paradigm towards exploiting readily accessible overexpressed nucleolin at the tumor vasculature, enabling cell internalization, provided a significant improvement in the intratumor bioavailability of the delivered drug, at a lower, but safer, systemic exposure than Caelyx/Doxil [19]. The referred-to effect was further translated into a marked nucleolin-dependent therapeutic efficacy, supported by a significant tumor growth inhibition of orthotopic mesothelioma tumors, along with a decrease in the nucleolin-positive vasculature density and downregulation of typically overexpressed genes in patients [19]. Importantly, an analysis of the breast and mesothelioma transcriptomic data from patients correlated nucleolin mRNA expression with prognosis, distinguishing biologically different tumors that may potentially benefit from PEGASEMP [19].

Building on the current state of the art, this work aimed at characterizing nucleolin expression in patient-derived pulmonary carcinomas and further validating nucleolin as a novel target to mediate successful therapeutic interventions against human lung cancers. In this respect, the activity of pH-sensitive liposomes containing doxorubicin and functionalized with the nucleolin-binding F3-peptide against lung cancer cell lines was further assessed.

## 2. Materials and Methods

### 2.1. Chemicals, Reagents and Drugs

Doxorubicin hydrochloride was purchased from IdisPharma (Weybridge, UK). Calcein, 4-(2-Hydroxyethyl)piperazine-1-ethanesulfonic acid (HEPES), 2-(N-Morpholino) ethanesulfonic acid (MES), disodium ethylenediaminetetraacetate dihydrate (EDTA), Trizma^®^Base, Sephadex G-50, ammonium sulfate, sodium chloride, 3β-hydroxy-5-cholestene-3-hemisuccinate (CHEMS), cholesterol (CHOL), and resazurin sodium salt were purchased from Sigma-Aldrich (St. Louis, MO, USA). The lipids 2-dioleoyl-sn-glycero-3-phosphoethanolamine (DOPE), 1,2-distearoyl-sn-glycero-3-phosphocholine (DSPC), 1,2-distearoyl-sn-glycero-3 phosphoethanolamine-N-[methoxy(polyethylene glycol)-2000] (DSPE-PEG_2k_), 1,2-distearoyl-sn-glycero-3-phosphoethanolamine-N-[maleimide(polyethylene glycol)-2000] (DSPE-PEG_2k_-maleimide), L-α-Phosphatidylethanolamine-N-(lissamine rhodamine B sulfonyl) (ammonium salt) (Egg-Transphosphatidylated, Chicken) (Egg Liss Rhod PE) were purchased from Avanti Polar Lipids (Alabaster, AL, USA). F3 (KDEPQRRSARLSAKPAPPKPEPKPKKAPAKK), and the non-specific (NS) (ARALPSQRSR) peptides [20] were custom synthesized by Genecust (Boynes, France).

### 2.2. Cell Lines

A549 (ATCC^®^ CCL-185™; CVCL_0023), H1975 (ATCC^®^ CRL-5908™; CVLC_1511), and H441 (ATCC^®^ HTB-174™; CVCL_1561) human lung cancer cell lines and the nucleolin-overexpressing MDA-MB-435S (ATCC^®^ HTB-129™; CVCL_0622) cell lines were cultured in RPMI 1640 medium (Sigma-Aldrich, St. Louis, MO, USA) supplemented with 10% fetal bovine serum (FBS, Alfagene, Carcavelos, Portugal) and 1% penicillin-streptomycin (PS, 100 U/mL, 100 µg/mL, Lonza, Basel, Switzerland) and maintained at 37 °C in a 5% CO_2_ atmosphere up to 1 month (from the original thaw) to prevent unwanted mutations. Non-tumorigenic MCF12A (ATCC^®^ CRL-10782™; CVCL_3744) cell line was cultured in RPMI 1640 medium (Sigma-Aldrich, St. Louis, MO, USA) supplemented with 5% FBS, 1% PS, human EGF (20 ng/mL, Sigma-Aldrich, St. Louis, MO, USA), and hydrocortisone (0.5 mg/mL, Sigma-Aldrich, St. Louis, MO, USA). Cell lines were regularly checked for the absence of mycoplasma infection, following the Center for Neuroscience and Cell Biology (CNC) internal rules. Cell lines authentication was performed by short tandem repeat profiling.

### 2.3. Analysis of Lung Cancer Datasets from the Cancer Genome Atlas

The lung adenocarcinoma (LUAD, *n* = 566) and squamous carcinoma (LUSC, *n* = 487) datasets from the Cancer Genome Atlas (TCGA; PanCancer Atlas) were accessed through the cBioPortal (http://www.cbioportal.org/, accessed on 1 February 2019). Only the cases reporting mRNA expression were considered and further filtered for the absence of clinical or time-to-event information. Overall, a total of 501 and 479 cases of lung adenocarcinoma and squamous carcinoma datasets were analyzed to estimate cohort parameters according to nucleolin expression. Nucleolin mRNA expression and patient survival data were plotted considering the best cutoff provided by survminer R package (v0.4.6) using maximally selected rank statistics [21]. Five-year statistics and median time-to-event were calculated with survival R package (v3.1-12). Further data visualization was created with Circos Online tool [22].

### 2.4. Validation of Nucleolin Expression in Pulmonary Carcinomas–Surgical Specimens

Fifty-eight human pulmonary carcinomas and adjacent non-neoplastic lung tissues collected from surgical specimens corresponding to 39 men and 19 women, averaging 66 years old, were selected both from the paraffin archives and as frozen samples collected prior to any treatment. Pulmonary carcinomas were classified according to the WHO 2015 criteria [3] after immunohistochemical characterization (panel composed by CK7, TTF-1, CK5/6, CD56, vimentin, and Ki67) as 28 adenocarcinomas (AD), 12 squamous cell carcinomas (SQ), 10 pleomorphic carcinomas (PM), and 8 adenosquamous carcinomas (ADSQ). Based on the UICC-TNM, there were 47 (81%) in stage levels I-IIB and 11 (19%) in stage levels III-IV. A total of 20 patients (34%) presented lymph node metastasis, while 38 (66%) were negative. Additional data were collected on the smoking status: 14 current smokers (24%) and 44 non-smokers and previous smokers (76%).

FFPE tumoral sections (3 µm thickness) were processed by standard methods and placed on coated slides (SuperFrost Ultra Plus^®^, Inopat, Porto, Portugal) and dried overnight at 37 °C for subsequent validation of Tumoral Endothelial Cells (TEC), Tumoral infiltrating Lymphocytes (TIL) and cancer-associated fibroblasts (CAF) based on cell morphology in hematoxylin-eosin slides (TEC = spindle-like within identifiable vessels; TIL = small, round; CAF = spindle-like) and immunochemical analysis of nucleolin. Lab Vision^TM^ UltraVision^TM^ LP Detection System (Thermo Fisher Scientific, Carlsbad, CA, USA) was used to increase sensitivity and detection of nucleolin, according to the manufacturer’s instructions. Briefly, sections were deparaffinized in xylene (twice, 10 min), rehydrated in absolute alcohol (twice, 5 min), rinsed in distilled water, and subjected to a heat-induced antigen retrieval (98 °C, 20 min). Sections were further incubated with 3% hydrogen peroxide for 15 min to block endogenous peroxidase activity, followed by 5 min incubation with Ultra V Block (TP-125-UB, UltraVision kit, Thermo Fisher Scientific, Carlsbad, CA, USA) at room temperature (RT) to block nonspecific binding, and incubation with mouse anti-nucleolin antibody (clone ZN004, 20 µg/mL, Thermo Fisher Scientific, Carlsbad, CA, USA) for 30 min at RT. Sections were then washed with phosphate-buffered saline (TP-125-PB, UltraVision kit, Thermo Fisher Scientific, Carlsbad, CA, USA) and incubated for 15 min at RT with the corresponding biotinylated goat anti-polyvalent secondary antibody (TP-125-BN, UltraVision kit, Thermo Fisher Scientific, Carlsbad, CA, USA). Upon washing, bound antibodies were visualized using peroxidase-conjugated streptavidin (TP-125-HR, UltraVision kit, Thermo Fisher Scientific) with 3,3′-diaminobenzidine tetrahydrochloride (DAB, RE7190-K, Novocastra Laboratories Ltd., Newcastle, UK) used as chromogen. Hematoxylin was used according to standard methods to counterstain the slides. Parallel known positive (nucleolin-positive human breast carcinoma tissue) and negative (nucleolin-positive without primary antibody incubation) controls were used.

Stained tumor sections were analyzed by two experienced pathologists in a blinded fashion using a Nikon H600L microscope equipped with a Digital Camera DXM 1200F (Nikon, Germany). Nucleolin expression was quantified based on the extent of staining (percentage of positive cells: 0–100%) and the degree of signal intensity (graded on a scale of 0–3: 0-low/negative; 1-weak; 2-moderate, and 3-high). A semi-quantitative global score was calculated multiplying the grades of both extent and intensity of staining and translated into a final score (Capuzzo’s) of 0–10% = negative; 11–100% = weak; 101–200% = moderate; and 201–300% = high applied to four high-power fields (×400) selected at random for each tumor specimen.

### 2.5. Preparation of Liposomes

Pegylated pH-sensitive liposomes, composed of DOPE:CHEMS:DSPC:CHOL:DSPE-PEG_2k_ at 4:2:2:2:0.8 molar ratio (in some experiments with 1 mol% RhoD-PE lipid (L-RhoD), relative to total lipid), with or without doxorubicin or calcein, were prepared by lipid film hydration method and further functionalized with the nucleolin-binding F3 peptide [18]. Briefly, dried lipid films were hydrated at 60 °C with 300 mM ammonium sulfate (pH 8.5) and the resulting liposomes were extruded through 80 nm pore size polycarbonate membranes using a LiposoFast Basic mini extruder (Avestin, Ottawa, ON, Canada). The buffer was exchanged in a Sephadex G-50 gel column equilibrated with 25 mM Trizma^®^Base in 10% sucrose solution (*w/v*, pH 9.0). Remote encapsulation of doxorubicin (18 mol% of doxorubicin relatively to total lipid), L[DOX]), was performed through the ammonium sulphate gradient method upon incubation with liposomes for 1.5 h at 60 °C [23]. Non-encapsulated doxorubicin was removed using a Sephadex G-50 gel column equilibrated with 25 mM HEPES, 140 mM NaCl buffer (HBS, pH 7.4).

To further prepare liposomes functionalized with the F3 peptide or a non-specific peptide, DSPE-PEG_2k_-peptide conjugate was synthesized. Briefly, thiolated derivative of the peptide was generated by reaction with 2-iminothiolane (Sigma-Aldrich, St. Louis, MO, USA) in 25 mM HEPES, 140 mM NaCl, 1 mM EDTA buffer (pH 8.0), for 1 h at room temperature, in an inert N_2_ atmosphere. Thiolated derivatives were then incubated overnight at room temperature with DSPE-PEG_2k_-maleimide micelles in 25 mM HEPES, 25 mM MES, 140 mM NaCl, 1 mM EDTA (pH 7.0). The resulting micelles of DSPE-PEG_2k_-peptide conjugates were post-inserted [24] onto the liposomal membrane at 2 mol% relative to total lipid (TL), upon incubation with pre-formed liposomes, for 1 h at 50 °C.

To prepare calcein-encapsulating liposomes (L-Calcein), the lipid film was hydrated with a 40 mM isosmotic calcein solution in HBS and extruded as described above. Following removal of non-encapsulated calcein through a Sephadex-G50 column equilibrated with HBS, liposomes were immediately submitted to the post-insertion procedure as previously described.

For the autoradiography studies, F3-peptide-targeted or non-targeted liposomes were labeled with ^99m^Tc-HMPAO as previously described [25].

Resulting mean size of prepared liposomes ranged between 70 and 90 ± 10 nm.

### 2.6. Association of ^99m^Tc-Labeled Liposomes with Patient-Derived Pulmonary Carcinoma Sections

Fresh lung tumor tissue after de-frost (initially rinsed in ice-cold saline and immediately snap-frozen in liquid nitrogen and stored at −80 °C) was sectioned (12–15 µm thickness and two adjacent slices *per* condition—one for autoradiography and the other for H&E analysis) at −20 °C, thaw-mounted onto SuperFrost Plus Microscope slides (Inopat, Porto, Portugal), and dried at 4 °C under negative pressure for 2 h.

The binding assay with ^99m^Tc-labelled liposomes was carried out as previously described [26], with slight modifications. Briefly, tumor sections were pre-incubated in 170 mM Tris-HCl buffer (Sigma-Aldrich, St. Louis, MO, USA) with 5 mM MgCl_2_ (pH 7.6) and 0.25% (*w/v*) bovine serum albumin (BSA) for 10 min at RT, and then incubated with ^99m^Tc-F3-L or ^99m^Tc-L (100 µCi in Tris-HCl buffer with 1% BSA) at RT for 1 h, and subsequently rinsed twice in cold Tris-HCl buffer (with and without 25% BSA) for 5 min, and in cold distilled water. Tumor sections were then air-dried and exposed overnight to a phosphor imaging screen (Kodak Phosphor Screen BAS-IP MS, GE Healthcare-Life Sciences, Marlborough, MA, USA). Images were revealed in a Typhoon FLA 9500 biomolecular imager (GE Healthcare-Life Sciences, Marlborough, MA, USA).

### 2.7. Subcellular Fractionation and Western Blotting

Four hundred thousand cells were washed twice with ice cold PBS, harvested, and sonicated in denaturing RIPA lysis (50 mM Tris, pH 8.0, 150 mM NaCl, 1% NP-40, 0.1% SDS, 0.5% sodium deoxycholate, 1 mM EDTA, 1 mM EGTA, and freshly added 1 mM DTT, 1 mM PMSF, protease, and phosphatase inhibitors, Sigma-Aldrich, St. Louis, MO, USA) and kept as whole cell samples. Nuclear and cytoplasmic fractionation was performed as previously described [27]. Briefly, one million cells were washed in PBS, dispersed, and lysed on ice for 30 min in subcellular fractionation (SF) buffer (20 mM HEPES, pH 7.4, 10 mM KCl, 2 mM MgCl_2_, 1 mM EDTA, and 1 mM EGTA, and freshly added 1 mM DTT, 1 mM PMSF, protease, and phosphatase inhibitors). Nuclei were sedimented by centrifugation (720 g for 5 min) and washed and dispersed twice in SF buffer to remove any contaminating cytoplasm, and then lysed and sonicated in RIPA buffer. The supernatants were centrifuged at 10,000 *g*, at 4 °C for 10 min, and further kept as the cytoplasmic fractions. Protein concentrations were determined by the BCA assay (Pierce, Chester, UK). Equal amounts of protein (7.5 µg) were denatured and boiled for 5 min, resolved on 10% sodium dodecyl sulfate (SDS)-polyacrylamide gels, and further transferred onto poly(vinylidene difluoride) (PVDF) membranes. These membranes were blocked for 1 h in Tris-buffered saline containing 0.1% Tween (*v/v*) and 5% non-fat milk (*w/v*) prior to incubation with primary antibodies. Immunoblotting was then performed by incubation overnight at 4 °C with the anti-nucleolin mouse antibody (clone EPR7952, 3.4 ng/mL, Abcam, Cambridge, UK), the mouse anti-GAPDH (clone GA1R, 0.5 µg/mL, Thermo Fisher Scientific), and the rabbit anti-LaminB1 (clone D9V6H, 1:500, Cell Signaling, Danvers, MA, USA) as loading controls, for the cytoplasmic and nuclear fractions, respectively, followed by 2 h incubation at RT with the corresponding AP-conjugated secondary antibodies (Invitrogen, part of Thermo Fisher Scientific, CA, USA). The immunoreactive bands were revealed by chemifluorescent reagent (Amersham Biosciences, Buckinghamshire, UK). Semi-quantitative analysis was carried out using ImageLab 4.1 image analysis software.

### 2.8. Quantification of Cell Surface Nucleolin

Cells were placed on ice and left undisturbed for 30 min, then centrifuged (170 g for 3 min) and resuspended in cold PBS buffer with 1% bovine serum albumin (PBS-BSA) and left undisturbed for 15 to 20 min. Cells were then centrifuged (170 g for 3 min) and resuspended in PBS-BSA containing 16.7 µM of DSPE-PEG-F3 micelles to prime nucleolin clustering at the cell surface [28], and 10 µg/mL mouse anti-nucleolin-Alexa^®^488 antibody (clone 364-5, lot GR314254-1, Abcam) or IgG1k isotype control (Affymetrix, Santa Clara, CA, USA) alone (as control), and placed in ice for 1 h. Cells were then washed twice and resuspended in PBS-BSA and transferred into cytometry tubes (BD Biosciences, San Jose, CA, USA) for flow cytometric quantification of nucleolin density at the cell surface in a BD FACSCalibur system (BD Biosciences, San Jose, CA, USA) using the Quantum^TM^ Alexa Fluor^®^ 488 MESF microspheres kit (lot 11488, Bangs Laboratories, Inc., Fishers, IN, USA). Briefly, the mean of cell surface nucleolin protein units per cell was calculated from the effective fluorescence to protein (F/P) ratio, i.e., the ratio of Alexa488 fluorescence in FL1 channel converted to molecules of equivalent soluble fluorochrome (MESF value) using QuickCal v2.3 (Bang Laboratories, Inc., Fishers, IN, USA) with the number of fluorochrome molecules attached to each antibody (dependent on the antibody batch) and assuming that each antibody bound to a single nucleolin protein (1:1 ratio). Non-viable cells were excluded using 7-aminoactinomycin D (7-AAD) (Sigma-Aldrich, St. Louis, MO, USA).

### 2.9. In Vitro Cellular Association, Internalization, and Cytotoxicity

For the cellular association studies, 200,000 lung cancer cells (from H1975, A549, and H441 cell lines) were seeded in 24-well culture plates for 24 h and then incubated with RhoD-labeled liposomes for 1 h, either at 4 (not permissive to endocytosis) or 37 °C. Cells were then washed with phosphate buffer saline (PBS, pH 7.4), detached with dissociation buffer (PBS, 1 mM EDTA), and immediately run in a FACSCalibur flow cytometer for detecting cell-associated rhodamine (FL2-H). A total of 20,000 events were collected and analyzed with CellQuest™ Pro software (Becton Dickinson, London, UK).

To assess liposomal internalization analysis, 200,000 cells (H1975 and A549) were seeded on a µ-slide 8-well ibiTreat plates (Ibidi, Gräfelfing, Germany) for 24 h and then incubated with calcein-labeled liposomes for 1 h at 37 °C. Cells were gently washed with Hanks’ Balanced Salt Solution (HBSS, Sigma-Aldrich, St. Louis, MO, USA), followed by lysosomal staining with 100 nM LysoTracker Red (Invitrogen, part of Thermo Fisher Scientific, CA, USA). After washing with HBSS at 37 °C, confocal images were acquired using a Confocal LSM 510 Meta microscope equipped with a Plan-Apochromat 63/1.4× Oil objective and an argon II (488 nm), a DPSS (561 nm), and a helium–neon (633 nm) excitation laser. Images were further analyzed with LSM 5 software (Zeiss, Oberkochen, Germany).

For cytotoxicity studies, six, seven, and eight thousand A549, H1975, and H441 cells, respectively, were seeded in 96-well plates and further incubated with serially diluted concentrations of doxorubicin, either free (from 0.01 to 2.50 µM) or encapsulated (at 0.39 to 100 µM) in non-targeted liposomes (L[Dox]) or functionalized by the F3 (F3-L[Dox]) or a control non-specific peptide (NS-L[Dox]), for 1, 4, or 24 h at 37 °C in a humidified 5% CO_2_ atmosphere. The medium was then replaced with fresh medium and the experiment was prolonged for a total of 96, 120, or 144 h, for A549, H1975, and H441 cells, respectively, as the result of their distinct proliferation rates (A549 > H1975 > H441), and thus enabling comparable doubling times. Cell viability was then evaluated by resazurin reduction assay at 570 (reduced form)–610 (oxidized form) nm in a Spectrophotometer SPECTRA max PLUS 384 (Molecular Devices, San Jose, CA, USA), as previously described [17]. IC_50_ and IC_90_ were calculated from the mean dose–response curves.

### 2.10. Statistical Analysis

Results were expressed as mean ± standard error of the mean (SEM). Statistical analysis was performed using adequate parametric (paired and unpaired) Student *t*-tests, and non-parametric (*Friedman*) and parametric *one-way* analysis of variance (ANOVA) followed by *Dunn’s*, *Dunnett’s*, and *Tukey’s* post hoc or the *Fisher’s* least significant difference (LSD) tests. Overall survival (OS) was estimated using Kaplan–Meier method. Univariate analysis for each prognostic variable was performed by using the log-rank test and expressed as mean ± standard deviation. Multivariate analysis was conducted using the Cox proportional hazards regression model to identify independent prognostic factors influencing OS. Significance thresholds were set at *p* < 0.05, *p* < 0.01, or *p* < 0.001, as defined in the text.

## 3. Results

### 3.1. Clinical and Prognostic Value of Nucleolin in Human Pulmonary Carcinomas

To determine the prognostic value of the nucleolin overexpression in human pulmonary carcinomas, the nucleolin mRNA expression in 501 lung adenocarcinomas and 481 lung squamous carcinomas from the TCGA were analyzed [29]. The lung adenocarcinomas presented a lower expression of nucleolin mRNA than lung squamous carcinomas (*p* < 0.0001, Figure S1A). Meanwhile, in the former, an increase in the nucleolin mRNA expression associated with advancing clinical stage was identified (*p* < 0.01, Figure S1B); such a relation was absent in the latter (*p* > 0.05, Figure S1B).

**Figure 1:**
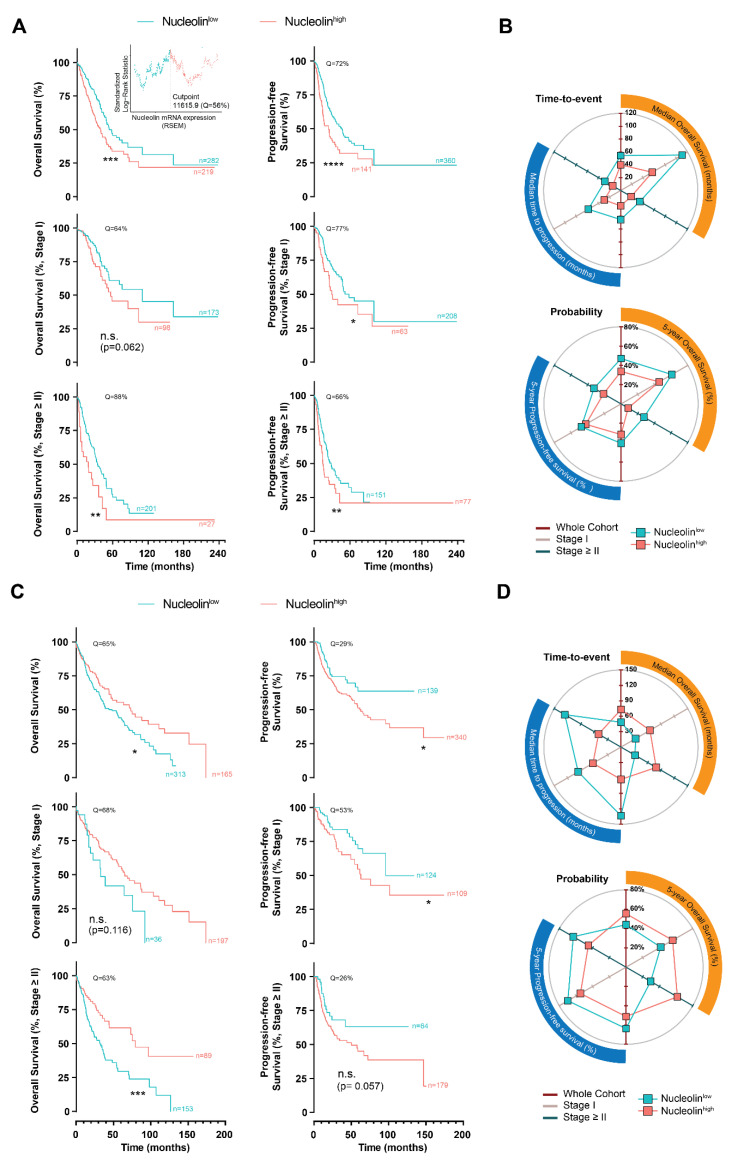
Epidemiological characterization of lung adenocarcinomas and squamous carcinomas as a function of nucleolin expression. TCGA’s lung adenocarcinomas and squamous carcinomas (PanCancer Atlas datasets, *n* = 566 and 487, respectively) were analyzed. Data were cleared from missing clinical information. (**A**–**D**) Time-to-event analysis (**A**,**C**) and respective median overall survival, time to progression, five-year overall, and progression-free survival (**B**,**D**) of patients from the lung adenocarcinoma (**A**,**B**) and squamous carcinoma (**C**,**D**) datasets, according to nucleolin mRNA levels (nucleolin^low^ and nucleolin^high^) and staging in primary tumors, stratified at identified quantiles (Q). Radar plots represented in (**B**,**D**) summarized the data presented in Tables S1 and S2: the closer to zero the points are, the worse the prognosis. The three axes (0–120% in Time-to-event; or 0–80% in Probability) represent the stratification of patients based on whole cohort (dark red axis), Stage I (light grey axis) and Stage ≥ II (dark blue axis). Insert in (**A**) demonstrates the determination of quantile cut-point by maximally selected rank statistics (**** *p* < 0.0001, *** *p* < 0.001, ** *p* < 0.01, * *p* < 0.05, n.s. *p* > 0.05, calculated by log-rank Mantel–Cox test). In (**D**), maximum time to progression is presented for nucleolin^low^ tumors from the whole cohort or with staging ≥II since median time could not be estimated).

Further stratification according to nucleolin expression levels (nucleolin^low^ and nucleolin^high^, below and above the indicated quantiles determined by maximally selected ranked statistics [21]) estimated a median OS over a year longer for nucleolin^low^ patients relative to nucleolin^high^ patients (54.3 months [95% CI: 49.1–110.5 months] and 39.9 months [95% CI: 31.3–50.2 months], respectively, *p* < 0.001), in the whole lung adenocarcinoma cohort (Figure 1A,B and Table S1). An analysis of the progression-free survival in the whole cohort correlated with the observation above, estimating more than 20 months longer median time to progression of patients presenting nucleolin^low^ lung adenocarcinomas compared to those nucleolin^high^ (45.3 months [95% CI: 35.6–62.2 months] and 23.9 months [95% CI: 15.5–34.4], respectively, *p* < 0.0001) (Figure 1A,B and Table S1). The progression-free benefit over 12 months identified for nucleolin^low^ patients, as compared to those nucleolin^high^, was sustained in the patients with lower (stage I, *p* < 0.05) or higher staging (stage ≥ II, *p* < 0.01), as well as in terms of survival in the case of the latter (*p* < 0.01, Figure 1A,B and Table S1).

Interestingly, the stratification of the patients with lung squamous carcinoma based on nucleolin expression inversely impacted OS, either in the whole cohort or under disease stage stratification (Figure 1C,D). In fact, the patients with nucleolin^high^ squamous carcinomas presented over 24 months longer median OS as compared with nucleolin^low^ tumors (73.1 months [95% CI: 46.9–118.4 months] vs 48.3 months [95%: 36.1–62.9 months], respectively) (Figure 1C,D and Table S1). Importantly, the analysis of the progression-free survival data from the squamous carcinoma dataset correlated with the observations in the lung adenocarcinoma (Figure 1B,D). Actually, it estimated a 1.5-fold longer median time to progression of nucleolin^low^ cases as compared with those nucleolin^high^, either in the whole cohort or in stage-controlled cohorts (Figure 1C,D and Table S1).

Altogether, the 5-year overall and progression-free survival correlated with the above results (Figure 1B,D and Table S2). Specifically, patients with nucleolin^low^ lung adenocarcinomas, with a staging ≥ II, presented a three-fold higher 5-year OS than those nucleolin^high^ (Figure 1B and Table S2). Conversely, and aligned with the above observations, lung squamous carcinomas overexpressing nucleolin presented a two-fold higher 5-year OS as compared to those nucleolin^low^ (Figure 1D and Table S2).

Further analysis of the patient distribution within staging and TMN grading as a function of nucleolin expression (based on OS data from whole cohorts) demonstrated that nucleolin^high^ lung adenocarcinoma cases are more prevalent (*p* < 0.01) at higher staging (50, 54, and 56% of the cases in Stage II, III, and IV, respectively), than in stage I (37% of cases) (Figure S2A). A similar trend was observed upon stratification by tumor grading, with nucleolin^high^ cases becoming more prevalent with increasing grading (38, 45, 52, and 68% of the cases classified as T1, T2, T3, and T4, respectively, *p* < 0.05) (Figure S2A), in line with previous observations (Figure S1). Even though a slight tendency in the same direction was observed in the case of metastasis and nodule grading, it was not statistically significant (Figure S2A). Regarding the lung squamous carcinoma dataset, nucleolin-expression-based stratification rendered a symmetric distribution of the cases along all the staging and TMN gradings (Figure S2B).

Overall, the data indicating the value of nucleolin as a predictor of survival prognosis may depend on the histological subtype of lung cancer. Yet, nucleolin mRNA overexpression may predict the disease progression of both human adeno and squamous pulmonary carcinomas.

### 3.2. Nucleolin Expression in Tumor Cells of Patient-Derived Pulmonary Carcinomas

**Figure 2:**
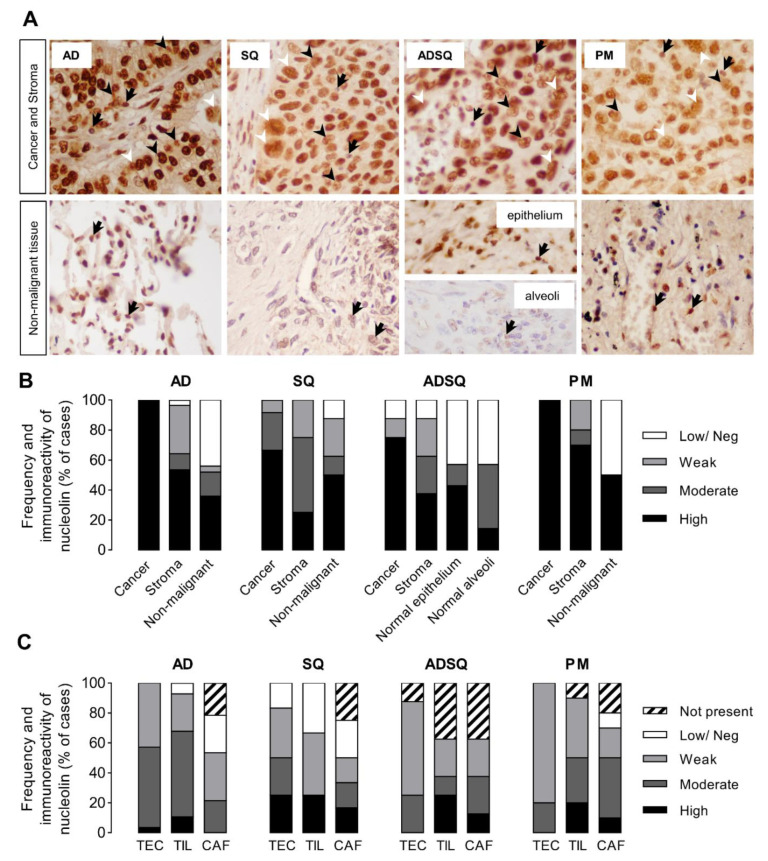
Nucleolin immunoreactivity among four histologically classified human pulmonary carcinomas (*n* = 58) and adjacent non-malignant lung tissues. (**A**) Representative images of the tumor cells (×400) and of the adjacent non-malignant tissues (×400) stained for H&E and immunostained for nucleolin. Arrows indicate nucleolar nucleolin; black arrowheads indicate nucleolin spreading throughout the nucleoplasm; white arrowheads indicate extra-nuclear nucleolin. See Supplementary Tables S3 and S4 for complete list of frequencies and global scores of nucleolin immunoreactivities (IR). (**B**) Prevalence of globally scored nucleolin (IR) (see Materials and Methods) as low/negative (white), weak (light gray), moderate (dark gray), and high (dark) in different sub-types of tumor (cancer and stromal) cells (adenocarcinoma, AD; squamous cell carcinoma, SQ; adenosquamous carcinoma, ADSQ; pleomorphic carcinoma, PM) and in the corresponding surrounding non-malignant tissues, as well as (**C**) in identifiable stromal cell types, namely tumor endothelial cells (TEC), tumor-infiltrating lymphocytes (TIL), and cancer-associated fusiform cells (CAFs).

Nucleolin is a ubiquitous nucleolar protein overexpressed in diverse human cancer tissues [30,31,32] and also overexpressed in the 58 patient-derived pulmonary carcinoma samples (Figure 2A,B, dark bars). Stromal cells presenting weak-to-high nucleolin immunoreactivity (light gray to dark bars, respectively) in all the histopathological types studied were also identified in adjacent non-tumoral lung tissues of the same origin (alveolar cells in adenocarcinoma, AD; respiratory epithelium in squamous cell, SQ and pleomorphic, PM; and mixed in adenosquamous, ADSQ; reviewed in Giangreco et al. [33]). A low/negative nucleolin expression was observed in 12.5 to 50% of the non-malignant samples. Interestingly, the nucleolin expression in non-malignant tissue (Figure 2A, arrows) was predominantly nucleolar, while the high intensity of nucleolin in cancer cells was redistributed throughout the nucleoplasm (black arrowheads) and extra-nuclear compartments (white arrowheads) in nearly 33% of all the cases, irrespective of the histological type and subtypes (see Table S3) and of other clinicopathological parameters, such as smoking status, TNM staging, and the presence of metastasis.

The tumor microenvironment is composed of heterogeneous cell populations playing a key role regarding tumor progression and initiation of metastasis, and, thus, they represent potential targets for nucleolin-based therapeutic intervention [34]. Therefore, the frequency of expression and levels of nucleolin immunoreactivity were further analyzed in stromal tumor endothelial cells (TEC), tumor-infiltrating lymphocytes (TIL), and cancer-associated fusiform cells (including fibroblasts, CAF). TEC and TIL were present in almost all cases (90–100%) and CAF in 75 to 80%, with an exception for ADSQ (TEC in 87.5% cases; TIL and CAF in 62.5% cases) (Figure 2C and Table S4). Additionally, more than 60% of the cases across the different subtypes showed mainly weak-to-moderate immunoreactivity for nucleolin (light gray to dark gray) in TEC, TIL, and CAF nuclear localization, except for SQ carcinomas (41.7%), whereas at least 17% to 33% of the cases showed low/negative nucleolin immunoreactivity. Together, these data underline increased levels of nucleolin in lung cancer cells from patient-derived tumors.

### 3.3. Ex Vivo Association of Nucleolin-Binding F3-Peptide-Targeted Liposomes with Patient-Derived Pulmonary Carcinomas

**Figure 3:**
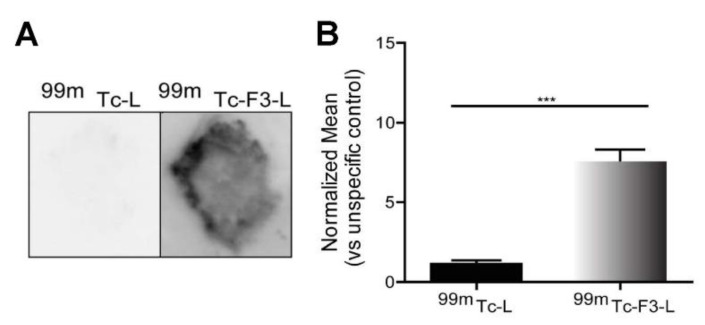
Ex vivo cellular association of radiolabeled nucleolin-binding F3-peptide-targeted liposomes to sections of patient-derived pulmonary carcinomas. Tumor sections were incubated for 1 h at room temperature with technetium-99m-labelled liposomes, either targeted with nucleolin-binding F3 peptide (^99m^Tc-F3-L) or non-targeted (^99m^Tc- L). (**A**) Representative autoradiograph images of bound liposomes to human pulmonary carcinomas and (**B**) the extent of binding is presented. Data represent the mean ± SEM, bars (n = 7), and were analyzed by two-tailed unpaired *t*-test (*** *p* < 0.001).

We further investigated whether the observed higher expression of nucleolin in human pulmonary carcinoma tissues is associated with its presence in the cell membrane, thus allowing different treatment modalities, such as intracellular drug delivery [18] or immunotherapy-based strategies [35]. Quantitative autoradiography of frozen human pulmonary carcinoma sections has shown that nucleolin-binding F3-peptide-targeted liposomes [18] radiolabeled with technetium−99m (^99m^Tc-F3-L) [25] presented a 5.6-fold higher association to human pulmonary carcinoma tissues than control non-targeted ^99m^Tc-liposomes (^99m^Tc-L) (Figure 3). This supported the presence of nucleolin at the outer surface of the cell membrane.

### 3.4. Nucleolin Is Present on the Cell Surface of Human Lung Cancer Cell Lines

**Figure 4:**
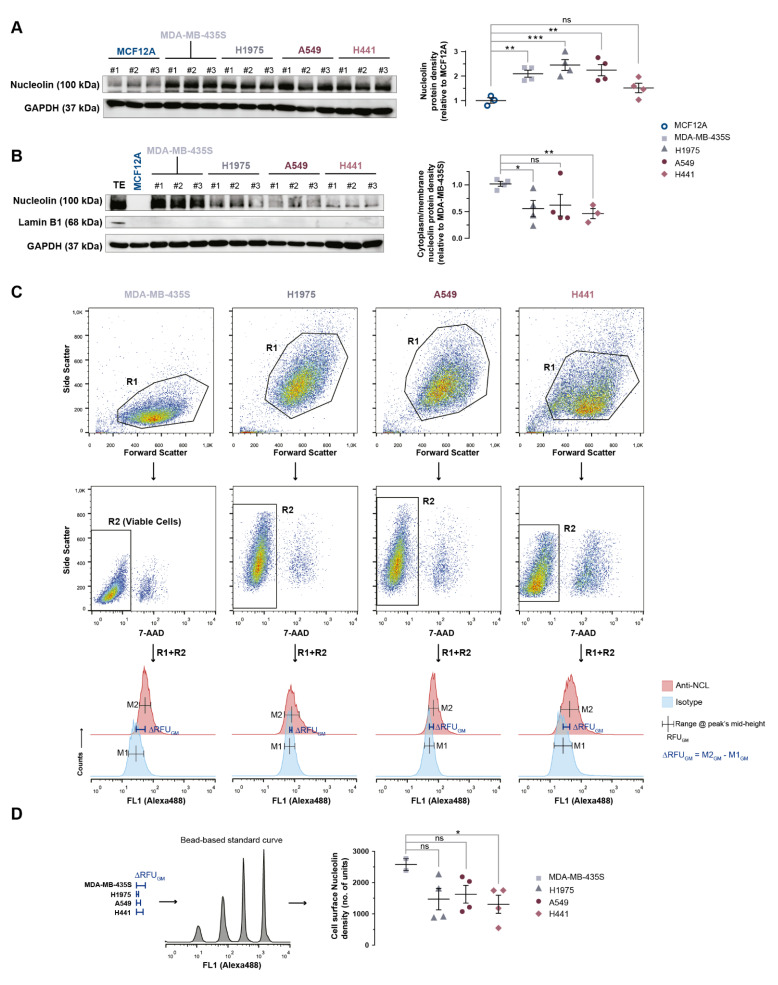
Density of nucleolin expression in lung cancer cell lines. Immunoblotting of nucleolin in total extracts (**A**) and in cytoplasm/membrane fractions (**B**) of non-small cell lung cancer H1975, A549, and H441, nucleolin-overexpressing MDA-MB-435S, and non-tumorigenic MCF12A cell lines. According to the condition, each lane represents an independent experiment. Data represent the mean ± SEM; total extracts were analyzed by 1-way ANOVA and Dunnett’s post hoc test (*n* = 3–4); cytoplasm/membrane extracts were analyzed by parametric unpaired *t*-test (*n* = 3–4). Whole Western blots can be found in Figure S3. (**C**) Representative dot plots and histograms associated with the strategy for analysis of cell surface nucleolin by flow cytometry. Dead cells were excluded using 7-actinoaminomycin D (7-AAD). (**D**) Quantification of cell surface nucleolin in the tumorigenic cell lines by flow cytometry. Data represent the mean ± SEM; *p*-value calculated with parametric unpaired *t*-test (*n* = 2–4). ns * *p* > 0.05, * *p* < 0.05; ** *p* < 0.01; *** *p* < 0.001.

The levels of expression of the nucleolin were assessed in human adenocarcinoma-derived lung cancer cell lines. In fact, the total nucleolin levels in the tested cancer cell lines were 2.4- (H1975; *p* < 0.05), 2.2- (A549; *p* < 0.05), and 1.5-fold (H441; *p* = 0.09) higher than in the negative control, the non-tumorigenic cell line MCF12A (Figure 4A). Similar levels were observed relative to the positive control nucleolin-overexpressing MDA-MB-435S cell line (*p* > 0.05; Figure 4A). Furthermore, nucleolin was also identified in the cytoplasm/membrane fractions (Lamin B1 negative) of all the lung cancer cell lines (Figure 4B), although to a lesser extent than in the MDA-MB-435S cell line (Figure 4B, H1975, 0.56 ± 0.15, *p* < 0.05; A549, 0.62 ± 0.40; *p* = 0.10; H441, 0.46 ± 0.16, *p* < 0.01), while it was undetectable in the non-tumorigenic cell line MCF12A (Figure 4B). An additional assessment of the surface levels of nucleolin by quantitative fluorescence flow cytometry confirmed the extent of expression (ranging from 51 to 63%) relative to the positive control of the nucleolin-overexpressing MDA-MB-435S cell line, which correlates (R^2^ = 0.99) with the corresponding densities in the cytoplasm/membrane fractions assessed by Western blot (Figure 4C,D).

Overall, these data support cell surface nucleolin as an attractive novel target for nucleolin-based therapeutic strategies as intracellular drug delivery systems in pulmonary carcinomas.

### 3.5. Significant Cellular Association and Internalization of Nucleolin-Binding F3-Peptide-Targeted Liposomes by Human Lung Cancer Cell Lines

Nucleolin-binding F3-peptide-targeted liposomes containing doxorubicin showed a significant in vitro intracellular delivery in both cancer (MDA-MB-435S) and endothelial cells (HMEC-1), relative to the non-targeted counterpart, and the ability to limit tumor invasion into adjacent healthy tissues in nucleolin-overexpressing MDA-MB-435S-derived mammary fat pad-implanted tumors [18]. Accordingly, and based on the results above, the ability of nucleolin to enable intracellular drug delivery into lung cancer cells (H1975, A549, and H441) was evaluated.

**Figure 5:**
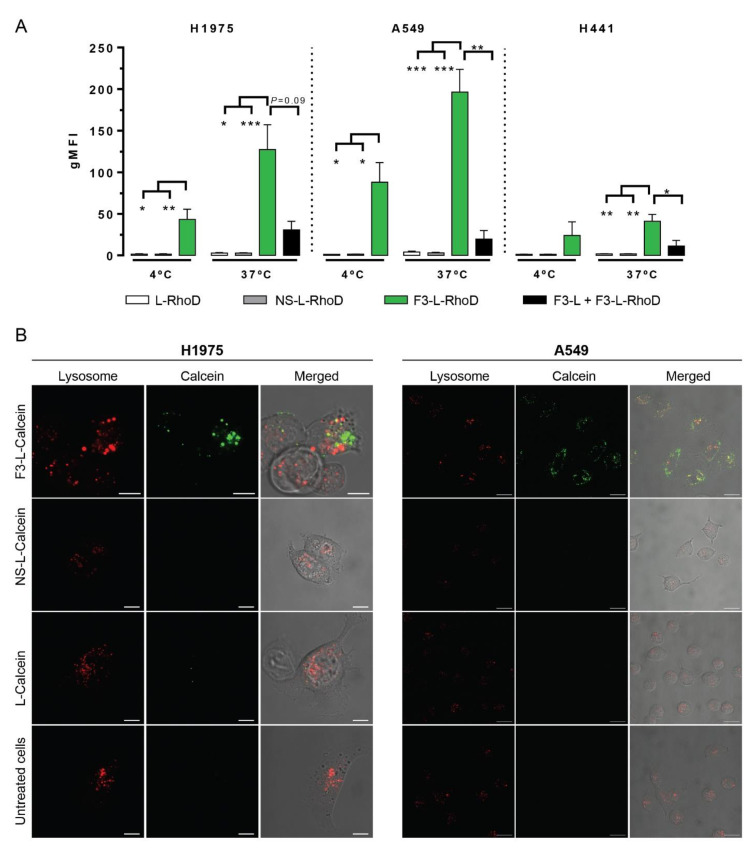
In vitro cellular association and internalization of nucleolin-binding F3-peptide-targeted and non-targeted liposomes by lung cancer cells. H1975, A549, and H441 lung cancer cells were left undisturbed (as control) or incubated at 4 °C (not permissive to internalization) or 37 °C, for 1 h, with rhodamine (Rhod) PE-labelled (**A**) or calcein-labelled (green, **B**, only at 37 °C) liposomes either non-functionalized (L) or functionalized with nucleolin-binding F3 peptide (F3-L) or non-specific control peptide (NS-L). (**A**) Results were expressed as the geometric mean value of the cell-associated fluorescence intensity (gMFI), normalized to untreated cells (*n* = 3–10), as assessed by flow cytometry. gMFIs of liposomal cellular association (F3-L-RhoD, green; NS-L-RhoD, grey; and L-RhoD, white) were represented as mean ± SEM and analyzed as matched data by *Friedman* test and *Dunn’s* post hoc test (for H1975) and by 1-way ANOVA and *Tukey’s* post hoc test (for A549 and H441; * *p* < 0.05; ** *p* < 0.01; *** *p* < 0.001); competitive inhibition study upon pre-incubation with F3-L (black) was compared to F3-L-RhoD, without pre-incubation (green), using paired *t*-test (* *p* < 0.05; ** *p* < 0.01). (**B**) Colocalization of calcein-labelled (green, aqueous core) liposomes (F3-L-Calcein, NS-L-Calcein, and L-Calcein) with Lysotracker Red (red, lysosomal marker) in H1975 and A549 cells using confocal microscopy. Scale bars: 10 µm (B, H1975) and 20 µm (B, A549).

We first performed cellular association studies of rhodamine PE-labelled pH-sensitive liposomes either non-functionalized (L-RhoD), or functionalized with nucleolin-binding F3 peptide (F3-L-RhoD) or a non-specific control peptide (NS-L-RhoD) at 4 °C (temperature non-permissive to endocytosis) or 37 °C for 1 h with the lung cancer cell lines. Cellular association of F3-L-RhoD (green bars) at 37 °C was 127- (H1975, *p* < 0.05), 197- (A549, *p* < 0.001), or 41-fold (H441, *p* < 0.01) higher than its non-targeted counterparts (NS-L-RhoD, grey bars; and L-RhoD, white bars) (Figure 5A) and notably highly correlated with cell surface nucleolin densities (Figure 4D, R^2^ = 0.94). Moreover, the 2.9- (H1975, *p* < 0.05), 2.2- (A549, *p* < 0.05), or 1.7-fold (H441, *p* = 0.34) difference relative to the corresponding experiments at 4 °C suggested an active endocytic liposomal internalization (Figure 5A). Furthermore, pre-incubation of cells with non-rhodamine labelled F3-peptide-targeted liposomes (F3-L) competitively enabled a high extent blockage of nucleolin binding sites that led to a marked reduction in the cellular association of F3-L-RhoD (Figure 5, black bars, H1975, *p* = 0.09; A549, *p* < 0.05; H441, *p* = 0.34).

Moreover, the intracellular green staining observed after incubation with F3-peptide-targeted liposomes loaded with calcein (F3-L-Calcein, green) supported a higher extent of nucleolin-mediated binding and intracellular delivery of the encapsulated payload into both cell lines relative to non-targeted liposomes (L-Calcein and NS-L-Calcein) and untreated cells, as assessed by confocal microscopy (Figure 5B). This result suggested an acidic pH-dependent liposomal destabilization, typical from an endocytic environment. This was supported by previous work from our group with F3-peptide-targeted liposomes formulated with 8 mol% of PEG, where encapsulated siRNA presented a marked co-localization with lysosomes [17]. The low prevalence of yellow staining suggested a reduced extent of co-localization between *Lysotracker Red* (red, a lysosomal marker) and calcein (green), ultimately indicating that the liposomal payload efficiently escaped from the endocytic route into the cytoplasm owing to an acidic destabilization of F3-peptide-targeted pH-sensitive liposomes [17,36]. Together, these data support the ability of nucleolin to enable intracellular drug delivery into lung cancer cells, in this case by nucleolin-binding F3-peptide targeted pH liposomes. The cellular dependency on intracellular delivery is reinforced by the absence of any differences in uptake between F3-peptide-targeted liposomes and a non-targeted counterpart by (non-cancer) BJ fibroblasts [17].

### 3.6. In Vitro Cytotoxicity of F3-Peptide-Targeted Liposomes Encapsulating Doxorubicin against Human Lung Cancer Cells

**Figure 6:**
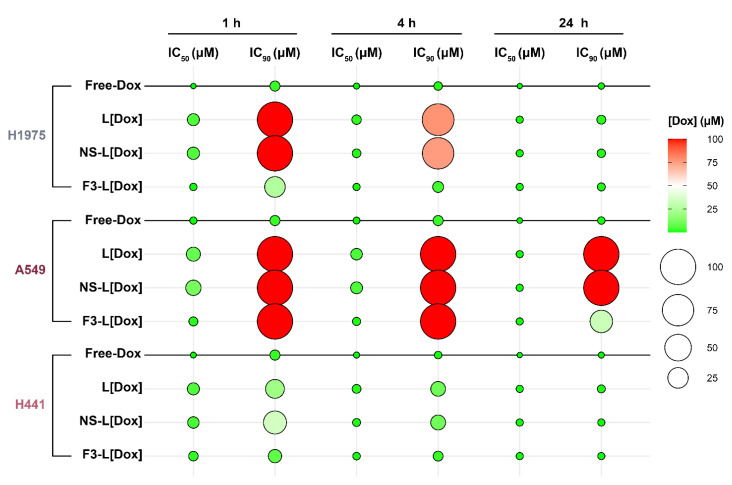
Cytotoxic potential of different formulations of liposomal doxorubicin against lung cancer cells. H1975, A549, and H441 lung cancer cells were incubated with increasing concentrations of doxorubicin (Dox), free or encapsulated in non-targeted (L[Dox]) or targeted pH-sensitive pegylated liposomes, functionalized either with a non-specific peptide (NS-L[Dox]) or nucleolin-binding F3 peptide (F3-L[Dox]), for the indicated periods. The medium was then replaced with fresh medium and the experiment was prolonged for a total of 96, 120, or 144 h, for A549, H1975, and H441 cells, respectively. Data represent the mean inhibitory concentration for 50% or 90% effect (IC_50_ and IC_90_, respectively). Circle size and color reflect the mean Dox concentration value (µM, *n* = 3): the smaller the size and the greener the color, the higher the cytotoxic potency. See Supplementary Table S5 for detailed data.

Upon the demonstration of nucleolin-mediated internalization of F3-peptide-targeted liposomes by lung cancer cell lines, the cytotoxic activity of encapsulated and free doxorubicin (used as a model of an anticancer drug) was assessed (Figure 6 and Table S5).

The overall low IC_50_ (50% inhibitory concentration) values of free doxorubicin for 1 h incubation supported the high sensitivity of all the tested cancer cells to the drug (Figure 6 and Table S5, H1975 > H441 > A549). Importantly, the delivery of doxorubicin by F3-peptide-targeted pH liposomes shifted the cell line drug sensitivity towards H1975 > H441 = A549 (*p* > 0.05). At 1 h incubation time, the IC_50_ values of F3-L[Dox] were lowered by approximately 12.4- (H1975; *p* < 0.05), 7.4- (A549; *p* < 0.05), and 2.1-fold (H441; *p* = 0.16) relative to its non-targeted counterparts (NS-L[Dox] and L[Dox]) (Figure 6 and Table S5). The corresponding IC_90_ values were also reduced at least by 3.9-fold in H1975 and by 2.9-fold in H441 (*p* < 0.05) (Figure 6 and Table S5).

Increasing the incubation time to 4 h dissipated the IC_50_ differences between F3-L[Dox] and the non-targeted counterparts to approximately 3.2- (H1975; *p* > 0.05), 5.4- (A549; *p* < 0.05), and 1.4-fold (H441; *p* > 0.05). A reduction in the IC_90_ was also observed, reaching values as low as 3.45 µM and 2.32 µM of doxorubicin in H1975 and in H441, respectively (22.5- and 4.2-fold lower concentrations relative to the non-targeted counterpart, respectively) (Figure 6 and Table S5). Interestingly, although we were unable to calculate IC_90_ values (within the range of concentrations used) upon 1 h and 4 h incubations of free- and liposomal-Dox in A549 cells, 24 h incubation with F3-L[Dox] showed a quantifiable 3.1-fold reduction in the IC_90_ value relative to the non-targeted counterparts (Figure 6 and Table S5).

Overall, the nucleolin-binding F3-peptide targeted liposomes (F3-L[Dox]) always demonstrated either improved cytotoxicity relative to non-targeted counterparts, regardless of the incubation time and the lung cancer cell line tested, or similar (*p* > 0.05) to free-Dox. This last result does not take into account the known unfavorable pharmacokinetics and biodistribution presented by the free drug in vivo [37].

## 4. Discussion

Herein, a cohort analysis of nucleolin mRNA expression in two TCGA datasets [29] (lung adenocarcinoma and squamous carcinoma) was performed in parallel with an immunochemical detection of the nucleolin levels in different histopathological subtypes of pulmonary carcinoma, surgically resected from patients, and in lung cancer cell lines. This was further complemented with a first assessment of the potential of nucleolin as a therapeutic target upon determining the in vitro activity of pegylated pH-sensitive liposomes containing doxorubicin and functionalized with the nucleolin-binding F3 peptide.

Nucleolin is a multifunctional phosphoprotein responsible for RNA regulatory mechanisms [9], thereby assuming a key role in cell survival and homeostasis. Its overexpression is constantly induced in exponentially growing cancer and endothelial cells [28,38], contributing towards uncontrollable growth and proliferation, overcoming of senescence, apoptosis, and immune system evasion, favoring invasion and metastization besides promoting angiogenesis [39].

Notwithstanding the multiple systematic meta-analysis that addressed the impact of Sox2 [40], nestin [41], and vimentin [42] as independent predictors in human lung cancer prognosis, two reports demonstrated the clinicopathological and prognostic significance of the nucleolin expression in lung cancer patients. Zhao et al. reported expression of nucleolin in endothelial cells (i.e., co-localized with CD31-positive vessels) in 34% of surgically resected lung squamous cell carcinoma and adenosquamous carcinoma patients. The combined score of its endothelial expression along with CD31, was identified as a possible prognostic factor of clinical outcome (poor survival) in these patients [38]. Furthermore, Xu et al. showed that expression of cytoplasmic nucleolin was associated with poor overall survival in lung adenocarcinoma and squamous cell carcinomas, whereas the nuclear protein was associated with better survival [43]. In accordance, high levels of nucleolin mRNA were predictive of poor prognosis and disease progression in the most common human pulmonary carcinoma subtype, i.e., lung adenocarcinoma, either on whole or stage-controlled cohorts (Figure 1A,B), in line with the correlation between nucleolin expression and staging (Figures S1 and S2). Interestingly, in lung squamous carcinoma, nucleolin overexpression only predicted disease progression, not overall survival (Figure 1C,D). In fact, lung adenocarcinoma and squamous carcinoma significantly differ in terms of the underlying biological processes. While in the former, processes related to cell division, including mitosis and RNA splicing are overly activated, in the latter, keratinization and keratinocyte differentiation are predominant [44]. Nucleolin has been shown to be overexpressed during the keratinocyte maturation, under Myc control [45]. Accordingly, in the case of lung squamous carcinoma, nucleolin overexpression may be related with a less aggressive, keratinocyte-enriched, phenotype as compared to a more undifferentiated (“more aggressive”) state of nucleolin^low^ tumors, in line with observations in breast cancer [46]. Alternatively, those differences may also be related with the inability of TCGA mRNA data to discriminate between nucleolin expression across tumor cell compartments, demonstrated to be important in a different lung cancer cohort [43]. Altogether, the patient prognostic and stratification value (relevant for nucleolin-based therapeutic interventions) of nucleolin mRNA expression should account for the histopathological subtype of lung cancer and further integrate an analysis at the protein level.

In fact, the immunohistochemical analysis of patient-derived pulmonary carcinomas showed strong nuclear and/or extra-nuclear (cytoplasm-membrane) nucleolin expression, in contrast with the diffuse nuclear staining in non-neoplastic adjacent tissues (Figure 2A,B). This is in line with previous observations in pulmonary carcinomas [43], hepatocellular carcinoma [30], and gastric carcinomas [32], which suggested a specific role for nucleolin at the cytoplasm and membrane subcellular localizations during lung carcinogenesis, yet to be determined, and as previously proposed for sirtuin 1 [47]. Moreover, the overexpression of nucleolin in cancer cells has been demonstrated to enable targeting towards cancer cells and the tumor microenvironment. Accordingly, the pH-sensitive liposomes containing doxorubicin and functionalized with the nucleolin-binding F3 peptide herein tested, upon intravenous administration, have significantly decreased the burden of tumors derived from nucleolin^high^ MDA-MB-435S cells, in contrast to tumors derived from nucleolin^low^ 4T1 cells, expressing 2.3-fold lower cell surface nucleolin relative to the former [19]. In the former tumors, the targeted liposomes enabled a 19-fold decrease in the nucleolin^+^ tumor vasculature density relative to a non-targeted liposomal formulation used as a control [19]. These studies have been complemented by a clear demonstration of therapeutic efficacy by the former formulation against different sub-types of nucleolin-overexpressing mesothelioma orthotopic tumors, relative to the standard of care, while presenting a safe profile, including hematological and clinical chemistry parameters as assessed in rats and dogs and in comparison with a non-targeted formulation [19].

As carcinomas are a complex and heterogeneous disease rather than characterized solely by malignant epithelial cells [34], the present histological examination of pulmonary carcinoma samples also explored the supportive stromal tumor tissue as its cellular compartment may represent a relevant target for nucleolin-based therapeutic intervention. In fact, most of the stromal cells morphologically identified, namely tumor endothelial cells, tumor-infiltrating lymphocytes (TIL), and cancer-associated fusiform cells (CAF), showed weak-to-moderate expression of nuclear nucleolin (Figure 2C). The malignant epithelial cells-stromal interactions and the associated activated stroma cells may determine specifically different clinical outcomes. While tumor endothelial cells are known to be responsible for tumor angiogenesis enabling cancer progression and metastasis [48], CAF encompass a heterogeneous subset of cells argued to exert a dual tumor promoter-suppressive action within the stromal compartment upon regulating angiogenesis and the recruitment and activation of immune cells [49]. For instance, distinct CAF molecular signatures can give rise to opposing outcomes in terms of tumor progression in pulmonary carcinomas [50,51], correlating, as well, with drug resistance [52]. Moreover, higher levels of TIL have been mostly associated with a better prognosis in pulmonary carcinomas [53,54], breast cancer [55], colorectal cancer [56], and liver metastasis [57]. In this respect, the present study provided additional evidence of nucleolin expression in stromal cells other than tumor endothelial cells, particularly in the nucleus (Figure 2). The latter pattern of expression in the overall tumor has been generally associated with a better prognosis relative to lower nuclear nucleolin expressors [31,32,43]. This unraveled a putative dual role for nucleolin within the tumor microenvironment, thus reinforcing the importance to clarify in the future the specific subcellular nucleolin functions and pattern of expression in all the cell compartments in both nucleolin^high^ and nucleolin^low^ tumors.

Notably, the association between increased mRNA nucleolin expression and more advanced disease stages (for lung adenocarcinoma, Figure S1), along with the prognostic value arising from its high expression in CD31-positive tumor endothelial cells (poor survival) [38], and in the cytoplasm/membrane (unfavorable survival) [32,43], emphasized the importance of gaining a better understanding of the overall role of nucleolin in lung cancer progression. In this respect, a link between nucleolin expression and the stem cell-like phenotype in breast cancer cells has been previously established [16]. In addition, cancer stem cell (CSC) markers, namely ALDH1 and CD133, have been linked with poor lung cancer prognosis [58,59]. As such, the reported association of nucleolin expression and poor prognosis in lung adenocarcinoma could indicate a potential role of this protein in lung CSC-mediated carcinogenesis [58,59]. Therefore, this would add a further dimension to nucleolin as a contributor for putative CSC-mediated intrinsic resistance to conventional lung cancer treatments [60,61], ultimately contributing to metastasis and recurrence. Hence, the high expression of nucleolin in a wide variety of lung cancer cells demonstrated in the present work, including stroma, along with its presence in the cell membrane, supported the relevance of the protein as a therapeutic target in the treatment of pulmonary carcinomas.

The therapeutic relevance of cell surface nucleolin as a path for intracellular drug delivery in pulmonary carcinomas has been further demonstrated as it associated with improved in vitro cytotoxicity. This was in agreement with the ability of cell surface nucleolin to mediate intracellular delivery of small peptides and proteins [11,28], siRNA [62], DNA [63], small molecular weight drugs [12,16], and extracellular vesicles [64] into multiple cancer cell types. In fact, the higher extent of association of liposomes targeting nucleolin relative to the non-targeted counterpart, evidenced by autoradiography in patient-derived tumor sections (Figure 3) and in lung cancer cell lines (by flow cytometry, Figure 5A), was likely due to cell surface expression of nucleolin, which further supported the original hypothesis that nucleolin qualifies as a therapeutic target in lung cancer. This was reinforced by Lai et al. upon the demonstration of lung tumor growth inhibition along with suppression of angiogenesis in a CL1-5 lung adenocarcinoma xenograft murine model (NOD-SCID) after tumor targeting with nucleolin aptamer-siRNA chimeras, which conveyed significant knockdown of snail family zinc finger 2 (SLUG) and neuropilin 1 (NRP1), key regulators of lung cancer cell metastasis and angiogenesis, respectively [63]. In this context, the present work explored the potential of pH-sensitive pegylated liposomes, functionalized with the nucleolin-binding F3 peptide [14,20] and containing doxorubicin, to enable intracellular delivery in lung cancer cells through cell surface nucleolin. Interestingly, the fold-change reduction in IC_50_ enabled by F3-L[Dox] at 1 h incubation positively correlated (R^2^ = 0.99) with the extent of its binding at 4 °C and with the cell surface levels of nucleolin (measured by flow cytometry) (Figure 4D and Figure 6 and Table S5).

## 5. Conclusions

Overall, the present study raises nucleolin as a relevant factor in lung carcinogenesis, defined by its overexpression in the tumoral microenvironment cell compartments and subcellular localization, ruling the time course of disease progression from the early to advanced stages. The potential of nucleolin as a therapeutic target is supported by its expression on the surface of the cells of tumors of diverse histopathological origins, such as hepatocellular carcinoma [65], neuroblastoma [66], gastric [67], and colorectal carcinomas [68], besides pulmonary carcinomas, as herein demonstrated. Together with the highlighted prognostic value of nucleolin, this work suggested the applicability of nucleolin-based targeting strategies against nucleolin^high^ pulmonary carcinomas, present at every disease stage, in a clinical trial setting.

## Data Availability

The data that support the findings of this study are available from the corresponding author upon reasonable request.

## Supplementary Materials

The following supporting information can be downloaded at: https://www.mdpi.com/article/10.3390/cancers14092217/s1, Figure S1: Nucleolin mRNA expression at different tumor stages of lung adenocarcinoma and squamous carcinoma; Figure S2: TCGA’s lung adenocarcinomas and squamous carcinomas (PanCancer Atlas datasets, *n* = 566 and 487, respectively) were analyzed; Figure S3: Whole Western blots; Table S1: Estimated median time-to-event for the lung adenocarcinoma (*n* = 501) and squamous carcinoma (*n* = 479) datasets from the TCGA; Table S2: Estimated 5-year overall and progression-free survival for the lung adenocarcinoma (*n* = 501) and squamous carcinoma (*n* = 479) datasets from the TCGA; Table S3: Global score of nucleolin immunoreactivity in different histologically classified human pulmonary carcinomas and adjacent non-malignant lung tissues (*n* = 58); Table S4: Global score of nucleolin immunoreactivity in tumor stromas of AD (*n* = 28), SQ (*n* = 12), ADSQ (*n* = 8), and PM (*n* = 10) human pulmonary carcinomas; Table S5: Cytotoxicity of different formulations of liposomal doxorubicin against lung cancer cells.
