# Supplementary material for: Nucleolin Overexpression Predicts Patient Prognosis While Providing a Framework for Targeted Therapeutic Intervention in Lung Cancer
Source: Cancers (Basel). 2022 Apr 29;14(9):2217. doi: 10.3390/cancers14092217 (PMC9101044; doi:10.3390/cancers14092217)
Supplement: Supplementary file 1 [file cancers-14-02217-s001.zip › cancers-1654932-supplementary.pdf]

cancers MDPI

Supplementary Materials

Nucleolin Overexpression Predicts Patient Prognosis While Providing a Framework for Targeted Therapeutic Intervention in Lung Cancer

Ângela Valério-Fernandes [1,2], Nuno A. Fonseca [1,3], Nélio Gonçalves [1], Ana F. Cruz [1,4], Marta I. Pereira [1,5], Ana C. Gregório [1,3], Vera Moura [1,3], Ana F. Ladeirinha [6], Ana Alarcão [6], Joana Gonçalves [7], Antero Abrunhosa [7], Joana B. Melo [8,9], Lina Carvalho [6], Sérgio Simões [1,4] and João N. Moreira [1,4,*]

Supplementary Figures

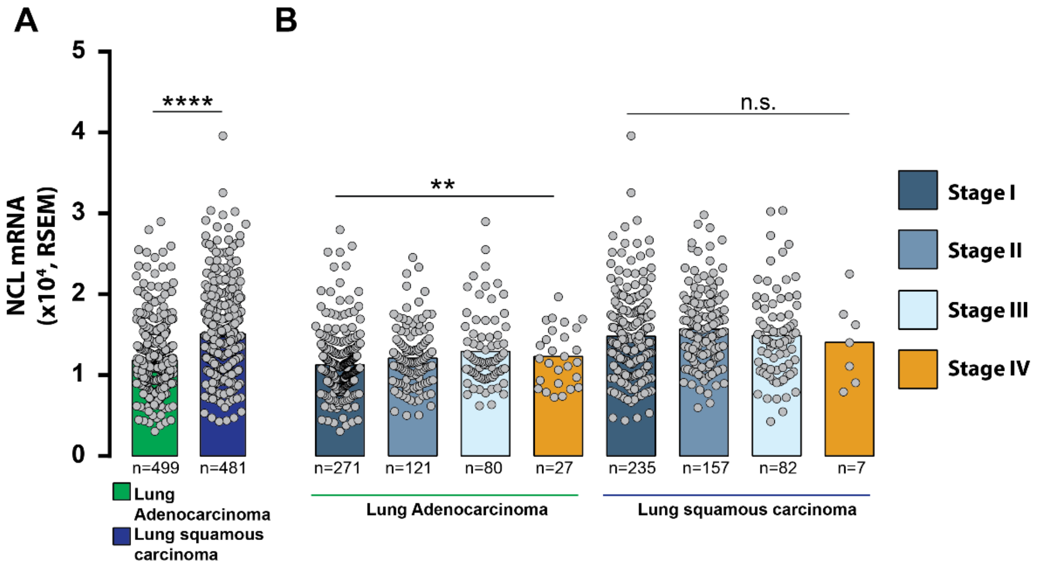

Figure S1. Nucleolin mRNA expression at different tumor stages of lung adenocarcinoma and squamous carcinoma. TCGA's lung adenocarcinomas and squamous carcinomas (PanCancer Atlas datasets, $n = 566$ and 487, respectively) were analyzed. Cases were selected according to completeness of available information. Representation of nucleolin mRNA expression levels (RNA-Seq by Expectation-Maximization–RSEM) (**A**) by lung adenocarcinomas or squamous carcinomas, (**B**) further stratified according to the pathological staging. Bars represent mean expression. Dots represent individual values (**** $p < 0.0001$ calculated by Mann-Whitney test; ** $p < 0.01$, $^{n.s.}p > 0.05$ calculated by Kruskal-Wallis test).

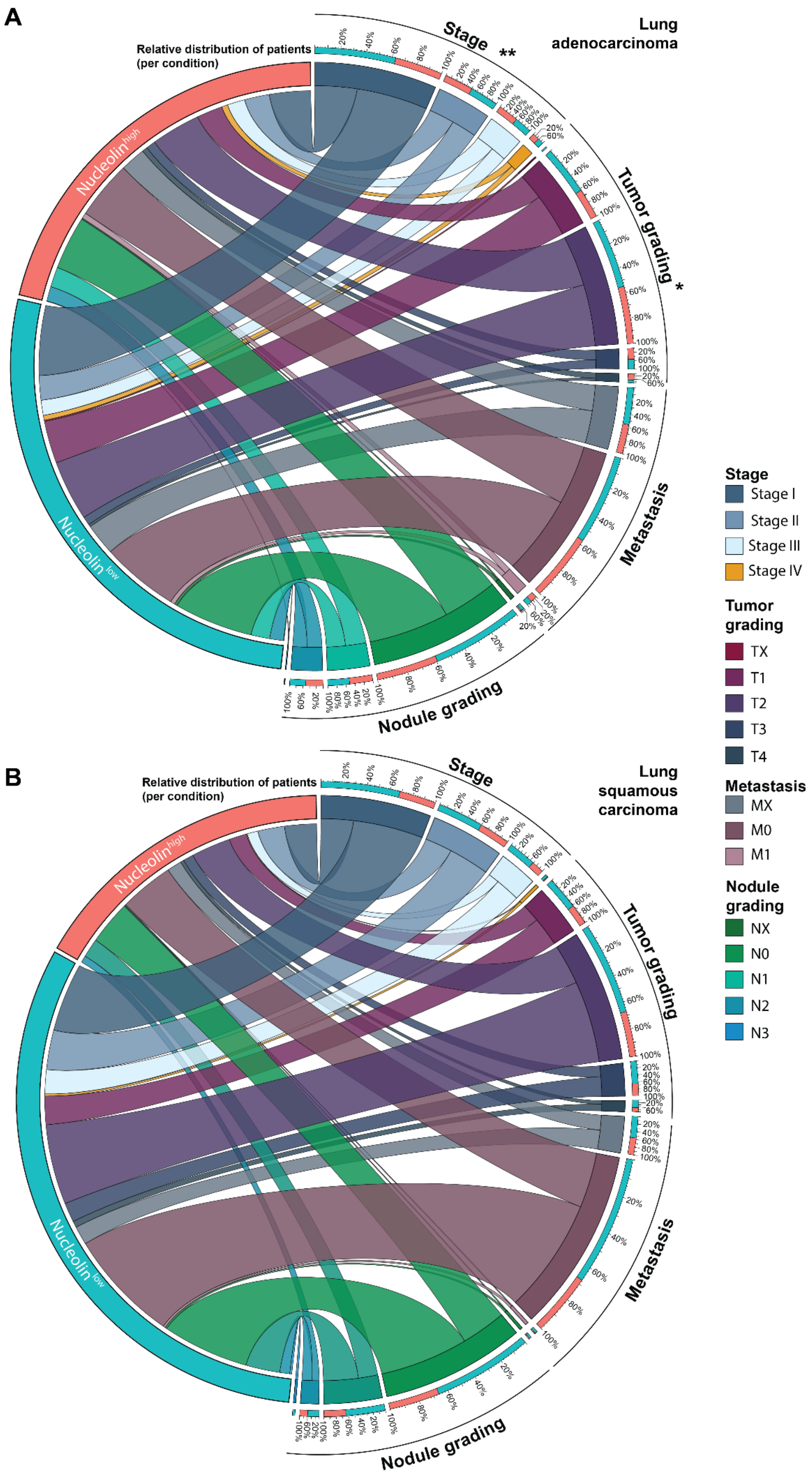
A
Relative distribution of patients (per condition)
Lung adenocarcinoma
Stage **
Tumor grading *
Metastasis
Nodule grading
Nucleolin high
Nucleolin low
B
Relative distribution of patients (per condition)
Lung squamous carcinoma
Stage
Tumor grading
Metastasis
Nodule grading
Nucleolin high
Nucleolin low
Stage
Stage I
Stage II
Stage III
Stage IV
Tumor grading
TX
T1
T2
T3
T4
Metastasis
MX
M0
M1
Nodule grading
NX
N0
N1
N2
N3

Figure S2. TCGA's lung adenocarcinomas and squamous carcinomas (PanCancer Atlas datasets, $n = 566$ and 487, respectively) were analyzed. **(A)** Lung adenocarcinoma and **(B)** squamous carcinoma grading characterization according to nucleolin expression levels (** $p < 0.01$, * $p < 0.05$, calculated by χ^2 test).

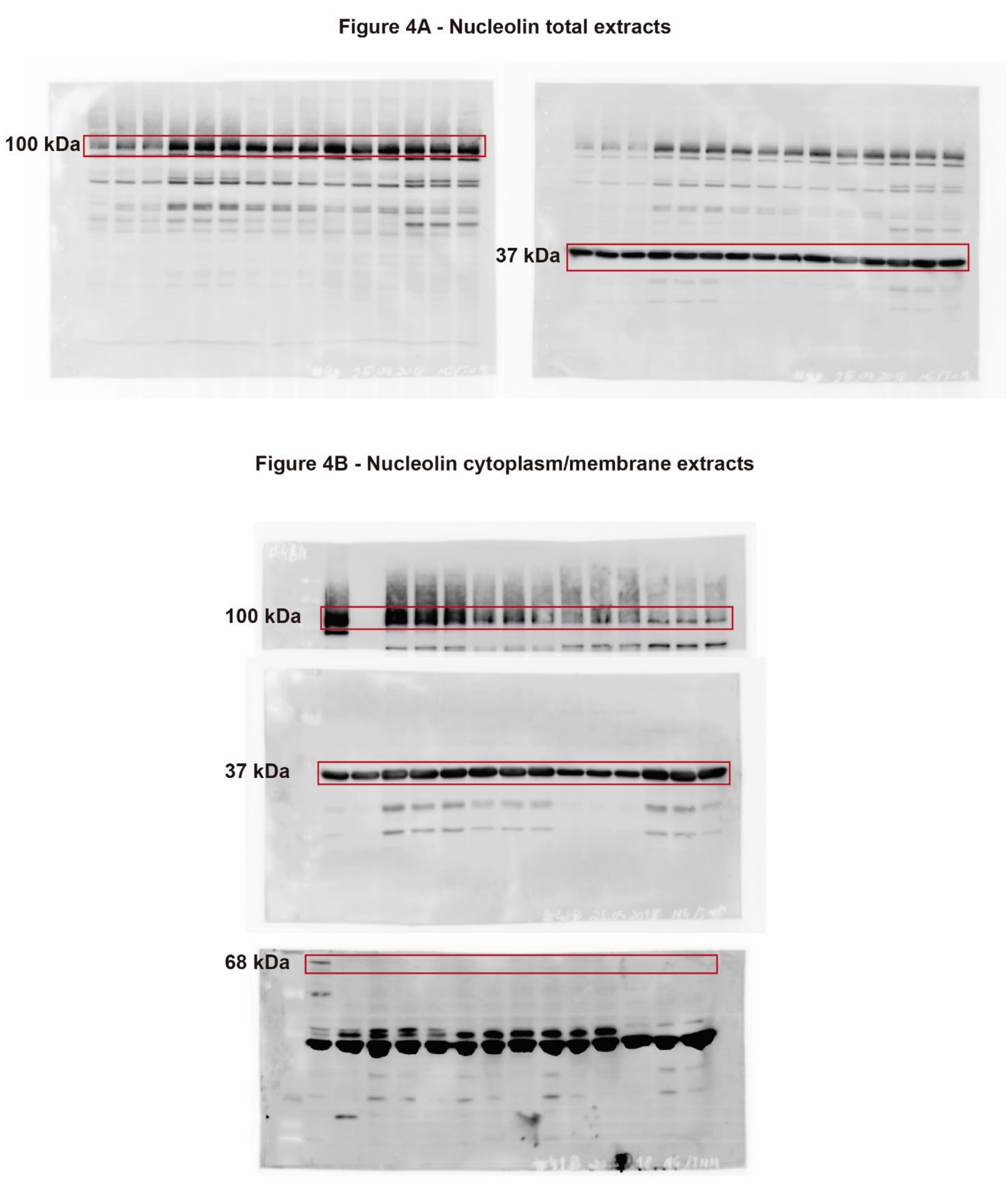

Figure S3. Whole western blots. Representative membranes stained with GAPDH (37 kDa); lamin B1 (68 kDa) or nucleolin (100 kDa).

Supplementary Tables

Table S1. Estimated median time-to-event for the lung adenocarcinoma (n=501) and squamous carcinoma (n=479) data sets from the TCGA

Disease	Parameter	Stage stratification	Nucleolin^{low}			Nucleolin^{high}		
			Median (months)	95% Confidence Interval		Median (months)	95% Confidence Interval	
				Lower	Upper		Lower	Upper
Lung Adenocarcinoma	Overall Survival	Whole Cohort	54.3	49.1	110.5	39.9	31.3	50.2
		Stage I	110.5	54.3	NA	56.7	42.3	NA
		Stage ≥ II	34.4	30.3	49.1	18.87	8.02	NA
	Progression-free survival	Whole Cohort	45.3	35.6	62.2	23.9	15.5	34.4
		Stage I	58.3	47.6	NA	29.5	25.4	NA
		Stage ≥ II	28.4	22.4	41.4	14.5	12.7	25.4
Lung squamous carcinoma	Overall Survival	Whole Cohort	48.3	36.1	62.9	73.1	46.9	118.4
		Stage I	32.9	19.3	NA	65.2	56.3	103.5
		Stage ≥ II	31.7	23.3	39.1	79.2	73.1	NA
	Progression-free survival	Whole Cohort	NA *	NA	NA	62.9	51.8	101.3
		Stage I	96.1	96.1	NA	62.9	54.4	NA
		Stage ≥ II	NA **	42	NA	50.3	26.4	NA

* Maximum: 133.7 months. ** Maximum: 126.2 months

Table S2. Estimated 5-year overall and progression-free survival for the lung adenocarcinoma (*n* = 501) and squamous carcinoma (*n* = 479) data sets from the TCGA.

Disease	Parameter	Stage stratification	Nucleolin^{low}			Nucleolin^{high}		
			5-year Survival (%)	95% Confidence Interval		5-year Survival (%)	95% Confidence Interval	
				Lower (%)	Upper (%)		Lower (%)	Upper (%)
Lung Adenocarcinoma	Overall Survival	Whole Cohort	47.1	38.2	58	33.7	25.4	44.9
		Stage I	60.9	49.8	74.5	45.6	32.5	63.9
		Stage ≥ II	27.5	18.9	40.2	8.53	1.43	50.9
	Progression-free survival	Whole Cohort	40.9	33.4	50	31.8	22.7	44.6
		Stage I	47.6	37.3	60.7	42.3	29	61.7
		Stage ≥ II	32.4	22.9	46	21	10.1	43.6
Lung squamous carcinoma	Overall Survival	Whole Cohort	44.1	37.2	52.2	55.5	46.4	66.3
		Stage I	41.6	24.3	71.5	55.9	47.4	65.9
		Stage ≥ II	29.6	20.6	42.5	61.5	50.1	75.6
	Progression-free survival	Whole Cohort	63.7	52.4	77.5	51.2	43.8	59.9
		Stage I	69.7	57.7	84.3	54.6	41.7	71.5
		Stage ≥ II	63.2	49.1	81.3	45.1	35.7	56.9

Table S3. Global score of nucleolin immunoreactivity in different histologically classified human pulmonary carcinomas and adjacent non-malignant lung tissues (*n* = 58).

Cell type	Nucleolin immunoreactivity	Histological type				
		Adenocarcinoma	Squamous cell carcinoma	Adenosquamous carcinoma	Pleomorphic carcinoma	Total
		Frequency of nucleolin expression (N, %)				
Cancer cells	Low/ Negative	0 (0.0)	0 (0.0)	1 (12.5)	0 (0.0)	1 (1.7)
	Weak	0 (0.0)	1 (8.3)	1 (12.5)	0 (0.0)	2 (3.4)
	Moderate	0 (0.0)	3 (25.0)	0 (0.0)	0 (0.0)	3 (5.2)
	High	28 (100.0)	8 (66.6)	6 (75.0)	10 (100.0)	52 (89.7)
	Extra-nuclear	9 (32.1)	4 (33.3)	2 (25.0)	4 (40.0)	19 (32.6)
Tumour stroma	Low/ Negative	1 (3.6)	0 (0.0)	1 (12.5)	0 (0.0)	2 (3.4)
	Weak	9 (32.1)	3 (25.0)	2 (25.0)	2 (20.0)	16 (27.6)
	Moderate	3 (10.7)	6 (50.0)	2 (25.0)	1 (10.0)	12 (20.7)
	High	15 (53.6)	3 (25.0)	3 (37.5)	7 (70.0)	28 (48.3)
Normal Lung Epithelium (Basal cells)	Low/ Negative	2 (8.0)	1 (12.5)	3 (42.9)	3 (50.0)	9 (19.6)
	Weak	3 (12.0)	2 (25.0)	0 (0.0)	0 (0.0)	5 (10.9)
	Moderate	2 (8.0)	1 (12.5)	1 (14.3)	0 (0.0)	4 (8.7)
	High	18 (72.0)	4 (50.0)	3 (42.9)	3 (50.0)	28 (60.9)
	n/a *	3	4	1	4	
Alveoli (Alveolar type II pneumocytes)	Low/ Negative	11 (44.0)	5 (55.5)	3 (42.9)	3 (33.3)	22 (44.0)
	Weak	1 (4.0)	1 (11.1)	0 (0.0)	2 (22.2)	4 (8.0)
	Moderate	4 (16.0)	2 (22.2)	3 (42.9)	3 (33.3)	12 (24.0)
	High	9 (36.0)	1 (11.1)	1 (14.3)	1 (11.1)	12 (24.0)
	n/a*	3	3	1	1	

* n/a: not applicable = either basal cells or type II pneumocytes were not present in the analyzed patient-derived adjacent non-malignant tissue of tumour slices.

Table S4. Global score of nucleolin immunoreactivity in tumor stromas of AD (n = 28), SQ (n = 12), ADSQ (n = 8) and PM (n = 10) human pulmonary carcinomas

Tumor Stroma		Frequency of Nucleolin Expression (N, %)			
**Histological type**	**Cell type (%#)**	**Negative**	**Weak**	**Moderate**	**High**
AD	TEC* (100)	0 (0.0)	12 (42.9)	15 (53.6)	1 (3.6)
	TIL** (100)	2 (7.1)	7 (25.0)	16 (57.1)	3 (10.7)
	CAF*** (78.6)	7 (25.0)	9 (32.1)	6 (21.4)	0 (0.0)
SQ	TEC (100)	2 (16.7)	4 (33.3)	3 (25.0)	3 (25.0)
	TIL (100)	4 (33.3)	5 (41.7)	0 (0.0)	3 (25.0)
	CAF (75.0)	3 (25.0)	2 (16.7)	2 (16.7)	2 (16.7)
ADSQ	TEC (87.5)	0 (0.0)	5 (62.5)	2 (25.0)	0 (0.0)
	TIL (62.5)	0 (0.0)	2 (25.0)	1 (12.5)	2 (25.0)
	CAF (62.5)	0 (0.0)	2 (25.0)	2 (25.0)	1 (12.5)
PM	TEC (100)	0 (0.0)	8 (80.0)	2 (20.0)	0 (0.0)
	TIL (90.0)	0 (0.0)	4 (40.0)	3 (30.0)	2 (20.0)
	CAF (80.0)	1 (10.0)	2 (20.0)	4 (40.0)	1 (10.0)

* Tumor Endothelial Cells; ** Tumor Infiltrating Lymphocytes; *** Cancer-Associated Fusiform cells/ Fibroblasts; # Percentage of cell type in the analyzed patient-derived tumor slices, for each sub-type.

Table S5. Cytotoxicity of different formulations of liposomal doxorubicin against lung cancer cells.

	**Compounds**	**1 h**		**4 h**		**24 h**	
		**IC_{50} (μM)**	**IC_{90} (μM)**	**IC_{50} (μM)**	**IC_{90} (μM)**	**IC_{50} (μM)**	**IC_{90} (μM)**
**H1975**	**free-Dox**	< 0.01	> 2.50	0.07 ± 0.01	1.11 ± 0.36	0.01 ± 0.00	0.13 ± 0.07
	**L[Dox]**	4.95 ± 1.10	> 100	1.85 ± 0.38	77.49 ± 42.36	< 0.39	1.42 ± 0.63
	**NS-L[Dox]**	5.44 ± 1.35	> 100	1.23 ± 0.10	75.48 ± 42.60	< 0.39	1.04 ± 0.75
	**F3-L[Dox]**	0.44 ± 0.25*	25.71 ± 12.94	< 0.39	3.45 ± 0.92	< 0.39	0.77 ± 0.49
**A549**	**free-Dox**	0.42 ± 0.00	> 2.50	0.27 ± 0.08	> 2.50	0.02 ± 0.00	0.51 ± 0.06
	**L[Dox]**	8.37 ± 0.89	> 100	4.30 ± 1.15	> 100	< 0.39	> 100
	**NS-L[Dox]**	11.10 ± 1.98	> 100	4.78 ± 1.25	> 100	< 0.39	> 100
	**F3-L[Dox]**	1.51 ± 0.79 *	> 100	0.89 ± 0.66*	> 100	< 0.39	32.51 ± 20.09
**H441**	**free-Dox**	0.04 ± 0.00	> 2.50	0.08 ± 0.03	0.54 ± 0.32	< 0.01	0.07 ± 0.06
	**L[Dox]**	5.03 ± 1.56	20.24 ± 3.62	1.34 ± 0.50	9.71 ± 3.56	< 0.39	0.77 ± 0.61
	**NS-L[Dox]**	4.15 ± 0.99	34.59 ± 11.36	0.79 ± 0.27	10.10 ± 5.79	< 0.39	< 0.39
	**F3-L[Dox]**	1.98 ± 0.79	7.09 ± 2.00#	0.56 ± 0.21	2.32 ± 0.82	< 0.39	< 0.39

Data represent the cytotoxic activities (IC_{50} and IC_{90}) from dose-response curves of either free or liposomal doxorubicin (n = 3). * F3-L[Dox] was compared with NS-L[Dox] and L[Dox] using one-way ANOVA and Tukey's post-test and differences were considered statistically significant with $P < 0.05$. # F3-L[Dox] was compared with L[Dox] using parametric unpaired t-test and difference was considered statistically significant with $P < 0.05$.
